# SAXS analysis of single- and multi-core iron oxide magnetic nanoparticles

**DOI:** 10.1107/S1600576717002370

**Published:** 2017-03-14

**Authors:** Wojciech Szczerba, Rocio Costo, Sabino Veintemillas-Verdaguer, Maria del Puerto Morales, Andreas F. Thünemann

**Affiliations:** a Federal Institute for Materials Research and Testing (BAM), Unter den Eichen 87, 12205 Berlin, Germany; b Academic Centre for Materials and Nanotechnology, AGH University of Science and Technology, Aleja Mickiewicza 30, 30-059 Kraków, Poland; c Instituto de Ciencia de Materiales de Madrid, ICMM/CSIC, Sor Juana Ines de la Cruz 3, 28049 Madrid, Spain

**Keywords:** superparamagnetic nanoparticles, iron oxide, reference materials, SAXS, small-angle X-ray scattering, XANES, X-ray absorption near-edge structure, X-ray absorption fine structure

## Abstract

This article reports on the characterization of four superparamagnetic iron oxide nanoparticles stabilized with dimercaptosuccinic acid, which are suitable candidates for reference materials for magnetic properties. Particles p_1_ and p_2_ are single-core particles, while p_3_ and p_4_ are multi-core particles. Small-angle X-ray scattering analysis reveals a lognormal type of size distribution for the iron oxide cores of the particles. Their mean radii are 6.9 nm (p_1_), 10.6 nm (p_2_), 5.5 nm (p_3_) and 4.1 nm (p_4_), with narrow relative distribution widths of 0.08, 0.13, 0.08 and 0.12. The cores are arranged as a clustered network in the form of dense mass fractals with a fractal dimension of 2.9 in the multi-core particles p_3_ and p_4_, but the cores are well separated from each other by a protecting organic shell. The radii of gyration of the mass fractals are 48 and 44 nm, and each network contains 117 and 186 primary particles, respectively. The radius distributions of the primary particle were confirmed with transmission electron microscopy. All particles contain purely maghemite, as shown by X-ray absorption fine structure spectroscopy.

## Abstract 2

Comprehensive characterization of the morphology of single-core and multi-core magnetic nanoparticle systems for future use as reference materials for magnetic properties was performed using a combination of small-angle X-ray scattering and static light scattering. The composition of the nanoparticle iron oxide cores was determined using XANES.

## 1. Introduction   

The synthesis, protection, functionalization and application of magnetic nanoparticles (MNPs) is a mature topic in nanochemistry (Lu *et al.*, 2007 ▸). Currently, the probably most attractive applications of MNPs lie in the biomedical field (Karimi *et al.*, 2013 ▸; Bohara *et al.*, 2016 ▸).

Vital for their practical realization is to standardize, improve and redefine the analytical methods pertaining to MNPs, as is currently underway in the European research project with the acronym NanoMag (Bogren *et al.*, 2105 ▸). To date there exists neither an MNP reference material nor a standard for defining properties of magnetic nanoparticles or characterization methods thereof. Although there are vendors on the market offering magnetic nanoparticle systems and labelling the magnetic properties with some numbers, these assertions cannot be verified since neither references nor standardized procedures exist to do so. Thus, a comparison of the product properties is virtually impossible for the customer. The situation for the characterization methods of magnetic properties of MNPs is no better. Results are strongly dependent on the experimental setup used, sample preparation and operation procedure, as well as the data reduction and analysis approach. The round robin studies performed so far are scarce and the results are deemed unpublishable. The situation would improve tremendously if there were a reference material addressing in a reproducible way at least one magnetic property, *e.g.* saturation magnetization, superparamagnetic blocking temperature or effective anisotropy constant. It is still under debate which of the many magnetic properties characterizes an MNP system most accurately for a specific application, but almost every one of them is related to the magnetic core size. One could relate the magnetic properties to energies instead of projecting them onto spheres of certain radii; this approach has not found its way into the MNP community and literature yet. Hence, the size of the magnetic cores and the distribution of core sizes remain among the most important characteristics of MNPs, which are used as an input for the determination and modelling of magnetic nanoparticles.

One can obtain the magnetic core sizes and their distribution from the analysis of magnetic data, for example, by analysing the high-field section of magnetization curves employing the Langevin function and assumed size distributions and shapes (Yoon, 2015 ▸). This information should be verified by complementary methods. Here, small-angle X-ray scattering (SAXS) is a natural solution for this problem. SAXS is capable of delivering representative and highly accurate data on the sizes of magnetic cores and on the distributions thereof. It provides incomparably more representative statistics on particle sizes and distributions than imaging techniques like transmission electron microscopy (TEM) or scanning electron microscopy, although those techniques are clearly better at determining the shapes of the cores. SAXS has a good reproducibility of the results, as the averaging depends on a physical effect rather than the judgement of the experimenter analysing an image and arbitrarily assigned particle size definitions.

Here, we report on a selection of four of the MNP systems under investigation, foreseen as reference materials for different magnetic properties that are designed to be biocompatible too. All four are stabilized with dimercaptosuccinic acid (DMSA) (Odio *et al.*, 2014 ▸). The ligand exchange process of oleic acid by DMSA results in MNP systems that are single-core particles (denoted p_1_ and p_2_) or display a multi-core structure (p_3_ and p_4_). An MNP reference material must have reproducible magnetic properties of interest. Since the magnetism of nanoparticles depends strongly on core sizes, shapes and crystalline structure, as well as aggregation behaviour, the reproducibility and reliable characterization of these parameters must be addressed first.

## 2. Experimental   

### 2.1. Synthesis of magnetic nanoparticles   

The procedure to obtain uniform magnetic nanoparticles with controlled core diameter by thermal decomposition and its transference to water takes place in three steps: (i) synthesis of the iron oleate precursor, (ii) synthesis of iron oxide magnetic nanoparticles and (iii) transference to water by ligand exchange. The synthesis was carried out following the procedure previously described by Salas *et al.* (2012 ▸) In brief, first, iron oleate was prepared by dissolving FeCl_3_·6H_2_O salt in water and adding it to a solution made of sodium oleate, ethanol and hexane. This mixture was heated at around 343 K for 4 h, washed with distilled water and ethanol in a funnel, and left to dry for two days. The iron content was 6%. Then, 4.5 g of the iron oleate was mixed with oleic acid in 50 ml of octadecene. The amount of oleic acid was varied between 0.6 and 1.2 g, leading to oleic acid/Fe molar ratios of around 4–3 to obtain particle sizes between 20 and 7 nm. The mixture was heated to 593 K at 3 K min^−1^. Finally, the particles were transferred to water by ligand exchange reaction with DMSA in a mixture of toluene and di­methyl­sulphoxide (Roca *et al.*, 2009 ▸). Applying this procedure, two single-core MNP systems, denoted as p_1_ and p_2_, and two multi-core MNP systems, p_3_ and p_4_, were produced. The production of single-core or multi-core particles depends on the efficiency of the ligand exchange process, which depends on the amount of oleic acid on the particle surface and the DMSA added (90 mg DMSA/50 mg Fe). In this work, the ligand exchange process was more efficient for the largest cores (>12 nm in diameter) owing to the reduction in specific surface area (lower amount of oleic acid on the nanoparticle surface). Larger amounts of DMSA (200 mg DMSA/50 mg Fe) are expected to lead to single-core particles for samples with smaller sizes (<10 nm in diameter).

### 2.2. SLS – static light scattering   

The SLS experiments were performed using a multi-angle detector setup equipped with an He–Ne laser from ALV, Langen, Germany. The samples were diluted by factors of 100, 200, 500, 1000 and 2000 to obtain a dilution series. The SLS data were converted to *I*(*q*) sets, by using the expression *q* = (4π*n*/λ)sinθ with λ = 632.8 nm (θ being half the scattering angle and *n* is the refractive index).

#### 2.2.1. SAXS measurements   

SAXS measurements were performed in a flow-through capillary with a Kratky-type instrument (SAXSess from Anton Paar, Austria) at 294 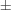 1 K. The SAXSess has a low sample-to-detector distance of 0.309 m, which is appropriate for investigation of dispersions with low scattering intensities. The samples were measured as delivered after vortexing for 3 min. The measured intensity was converted to an absolute scale according to Orthaber *et al.* (2000 ▸). The scattering vector magnitude *q* depends on the wavelength λ of the radiation (λ = 0.154 nm) as *q* = (4π*n*/λ)sinθ. Deconvolution (slit length desmearing) of the SAXS curves was performed with the *SAXS-Quant* software (Anton Paar). Samples analysed with SAXS were used as prepared. Curve fitting was conducted with the software *SASfit* (Bressler *et al.*, 2015 ▸).

### 2.3. XANES – X-ray absorption near-edge structure   

The X-ray absorption fine structure (XAFS) experiments were carried out at the BAMline (Görner *et al.*, 2001 ▸; Riesemeier *et al.*, 2005 ▸) with the BESSY II synchrotron light source in Berlin, Germany. The XAFS spectra at the *K* edge of iron (7112 eV) were recorded in transmission mode using two ionization chambers (Oxford Danfysik IC Plus 50). The incident beam intensity was monitored using an ionization chamber filled with air at ambient pressure, giving a transmission rate of 0.92 at 7100 eV. The absorption signal was measured using a second ionization chamber of the same type, filled with argon gas at ambient pressure, with a transmission rate of approximately 0.25 in the energy range of interest. The energy was scanned using an Si(111) double-crystal monochromator with a relative energy resolution of 5 × 10^−5^. The XAFS scans in the near-edge region (XANES) were carried out in the range from 7032 eV (80 eV below the edge) to 7182 eV (70 eV above the edge) with a step of 1 eV. The edge jump was measured with a finer step of 0.5 eV. The extended absorption fine structure region (EXAFS) was scanned up to *k*_max_ = 12 Å^−1^ above the absorption edge with a large step in the momentum space to make the normalization of the spectra more convenient. The scans were recorded with an acquisition time of 4 s per point. The spectra were calibrated to the absorption edge energy of metallic iron, 7112 eV. The energy step in the XANES region was smaller than the natural broadening caused by the finite mean lifetime of the excitation states.

### 2.4. TEM imaging   

Particle sizes and shapes were studied by TEM using a JEOL JEM 1010 microscope operated at 100 keV. TEM samples were prepared by placing one drop of a dilute particle suspension on an amorphous carbon-coated copper grid and evaporating the solvent at room temperature. The mean particle size and distribution were evaluated by measuring the largest internal dimension of at least 100 particles. Afterwards, data were fitted to a lognormal distribution, obtaining the mean size (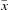) and the standard deviation (σ).

## 3. Results and discussion   

The spherical magnetic nanoparticle systems under investigation can be divided into two groups according to their coarse morphology. These are single-core and multi-core particles (Lartigue *et al.*, 2012 ▸). The single-core particles consist of isolated cores with functionalized coatings, whereas the multi-core particles are composed of cores that strongly interact with each other within agglomerates. When interpreting their magnetic properties, we regard these agglomerates of interacting cores as single magnetic nanoparticles. The particles denoted p_1_ and p_2_ are single-core particles, whereas p_3_ and p_4_ are multi-core ones judging by the routes of syntheses chosen by manufacturers and the available TEM data.

Here, SAXS was employed to determine the sizes of the particle cores and the distribution thereof. The intensity of the SAXS signal is proportional to the square of the density difference between the solvent and the object of interest. The density of Fe_2_O_3_ is approximately 4.9 g cm^−3^, while the density of DMSA is about 1.6 g cm^−3^. The solvent is water with 1.0 g cm^−3^. The contrast is the ratio of these squared differences (3.9^2^/0.6^2^). Hence, the iron oxide cores scatter X-rays about 50 times stronger than the organic shell made of dimercaptosuccinic acid. Therefore, we ‘see’ only the iron oxide with SAXS. The scattering intensity, 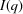, of particles in the monodisperse approximation is given as the product of the particle form factor, 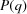, and the structure factor, 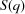, as
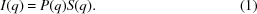
In this work we model the single-core particles with 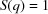, which means that no interaction is present between the particles. In contrast, the structure factor of the multi-core particle is 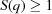.

## 4. Single-core particles   

**Figure 1:**
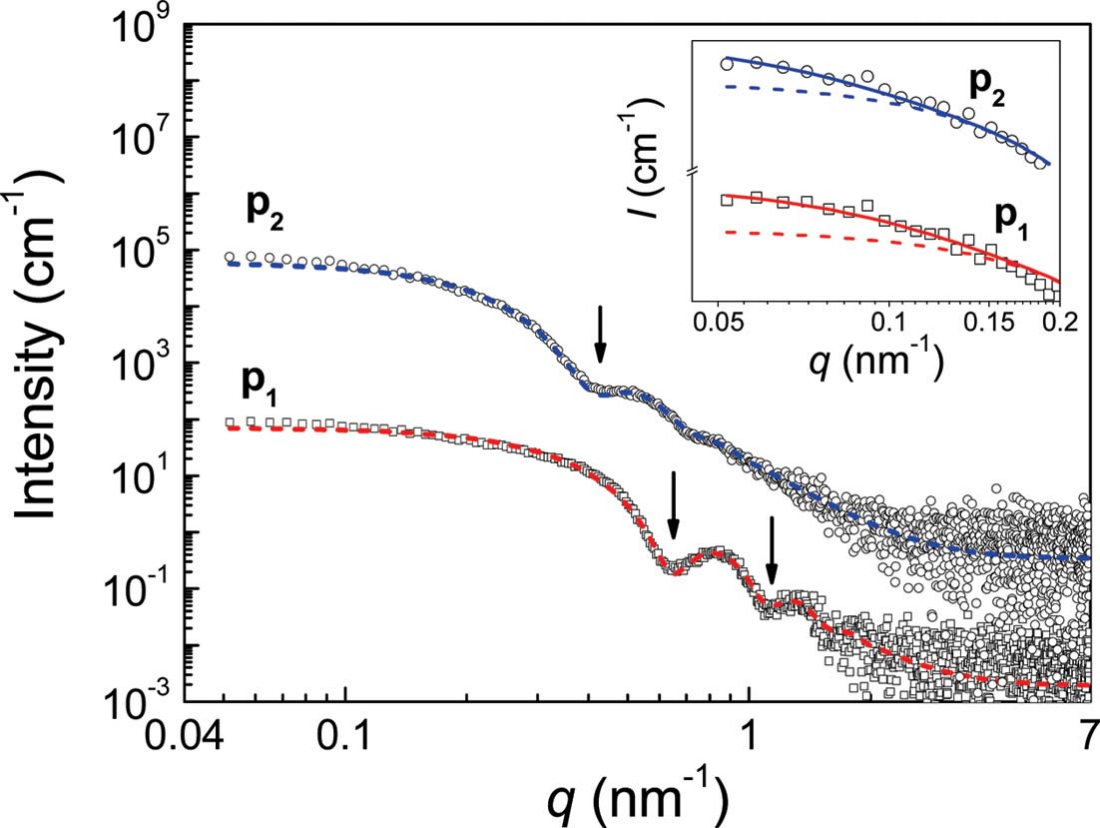
SAXS curve of single-core particles p_1_ and p_2_ (squares and circles, respectively). Data of p_2_ are multiplied by 10^3^ for better visibility. Curve fits utilizing a lognormal size distribution of the radii are given (red and blue dashed lines, respectively). Minima at *q* = 0.64 nm^−1^ and *q* = 1.11 nm^−1^ for p_1_ and at *q* = 0.42 nm^−1^ for p_2_ are indicated by arrows. Inset. Enlargement of the low-*q* range with addition of the scattering contribution of the small fraction of aggregates (solid curves).

The SAXS curve of p_1_ displays characteristically sharp minima at *q* = 0.64 nm^−1^ and *q* = 1.11 nm^−1^, which is a typical indication of particles with a very narrow size distribution (Bonini *et al.*, 2007 ▸). The scattering fringes of the SAXS curve of p_2_ are less pronounced than those for p_1_. Hence, the relative size distribution of the particle cores is broader. The first minimum at *q* = 0.42 nm^−1^ sets in at a lower *q* value than for p_1_, indicating that the particle cores of p_2_ are larger than those of p_1_ (see circles and squares, respectively, in Fig. 1 ▸). We found that the scattering curves are reproduced well using the analytical form factor of a sphere, 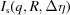, with a lognormal number-weighted size distribution of the radii [the use of this distribution function is justified in the supporting information; for further literature relating to this material, see O’Grady & Bradbury (1983 ▸) and Hamilton (1965 ▸)], 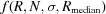, as
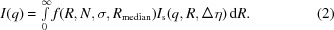
The lognormal size distribution is defined as
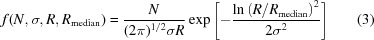
and the scattering of a single sphere is given by
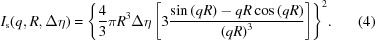

*R* is the sphere radius and Δη the scattering length density difference between the particle and the matrix. For iron oxides in aqueous solution Δη is 2.8974 × 10^−11^ cm^−2^ when presuming an Fe_2_O_3_ stoichiometry, a density of 4.9 g cm^−3^ and an X-ray energy of 8.6 keV. σ is the width parameter of the size distribution and *R*_median_ is the median radius. *N* is the particle number density. Using these parameters, the mean radius was calculated as 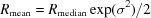 and the relative standard deviation of the width of the size distributions as 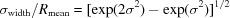.

**Figure 2:**
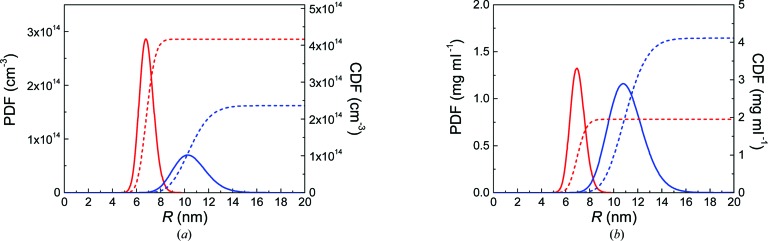
Size distributions of p_1_ and p_2_ (red and blue lines) in number-weighted and volume-weighted form [(*a*) and (*b*), respectively]. Displayed are the partial differential functions (solid lines) and cumulative distribution functions (dashed lines). The number-weighted distributions are given in units of particles per cm^3^. The volume-weighted size distributions in (*b*) are given in units of iron content in mg per ml, calculated from the particles’ volume fraction, a particle density of 4.9 g cm^−3^ and an Fe_2_O_3_ stoichiometry.

Equation (1) was employed for interpretation of the scattering data of p_1_ and p_2_, resulting in the fitted curves shown in Fig. 1 ▸ (red and blue dashed lines, respectively). The distributions of the particles’ number density in particles per cm^3^ as a probability density function (PDF) and a cumulative density function (CDF) are shown in Fig. 2 ▸(*a*) (solid and dashed curves, respectively). We calculated the volume-weighted PDF and CDF in terms of iron concentration for comparison (Fig. 2 ▸ *b*, solid and dashed curves). The mean radii are *R*_mean_ = 6.9 ± 0.1 nm (p_1_) and 10.6 ± 0.1 nm (p_2_), and the relative distribution width are 0.08 ± 0.01 and 0.13 ± 0.02. Additionally, the total particle number densities are *N* = (4.2 ± 0.2) × 10^14^ cm^−3^ and *N* = (2.4 ± 0.2) × 10^14^ cm^−3^ (see Fig. 2 ▸ *a*). Calculation of the volume fraction for p_1_ results in φ = (5.6 ± 0.2) × 10^−4^, which corresponds to a total iron oxide concentration of *c* = 2.7 ± 0.1 mg ml^−1^ and a total iron content of 1.9 ± 0.1 mg ml^−1^. Similarly, the volume fraction of p_2_ is φ = (1.1 ± 0.1) × 10^−3^, corresponding to a total iron oxide concentration of *c* = 5.6 ± 0.2 mg ml^−1^ and a total iron content of 4.0 ± 0.2 mg ml^−1^.

The low-*q* range of the SAXS curves exhibits a plateau toward zero, indicating that there are hardly any particles or agglomerates present in the sample that are significantly larger than the primary particle cores. However, the model fit with the primary particles for p_1_ very slightly underestimates the intensity of the experimental curve at *q* < 0.1 nm^−1^. The addition of a small population of larger spherical particles corrects this mismatch (see inset of Fig. 1 ▸). The spheres have a mean radius of about 21 nm and a particle number density of about 2 × 10^11^ cm^−3^. We interpret the larger structures as aggregates consisting of the smaller particles. The volume ratio indicates that there are around 30 primary particles contained in one aggregate on average. Furthermore, from the fitted intensity coefficients one can state that there are 2000 times more primary particles than aggregates. Most probably the cores are in direct contact within the aggregate and therefore SAXS cannot resolve the inner structure of these.

As in the case of p_1_, there is a small mismatch of the intensities of p_2_ between the model and the experiment at *q* < 0.1 nm^−1^. The addition of a small population of larger spheres corrects the underestimation in this case, too. Like for p_1_ we interpret this as the presence of a few aggregates consisting of the primary particle cores. The aggregates have a mean radius of about 28 nm. The particle number density is about 4 × 10^11^ cm^−3^. This means that one aggregate consists of approximately 20 primary particles. The ratio of the fitted intensity factors for the two populations suggests that there is one aggregate per 540 primary particles.

## 5. Multi-core particles   

**Figure 3:**
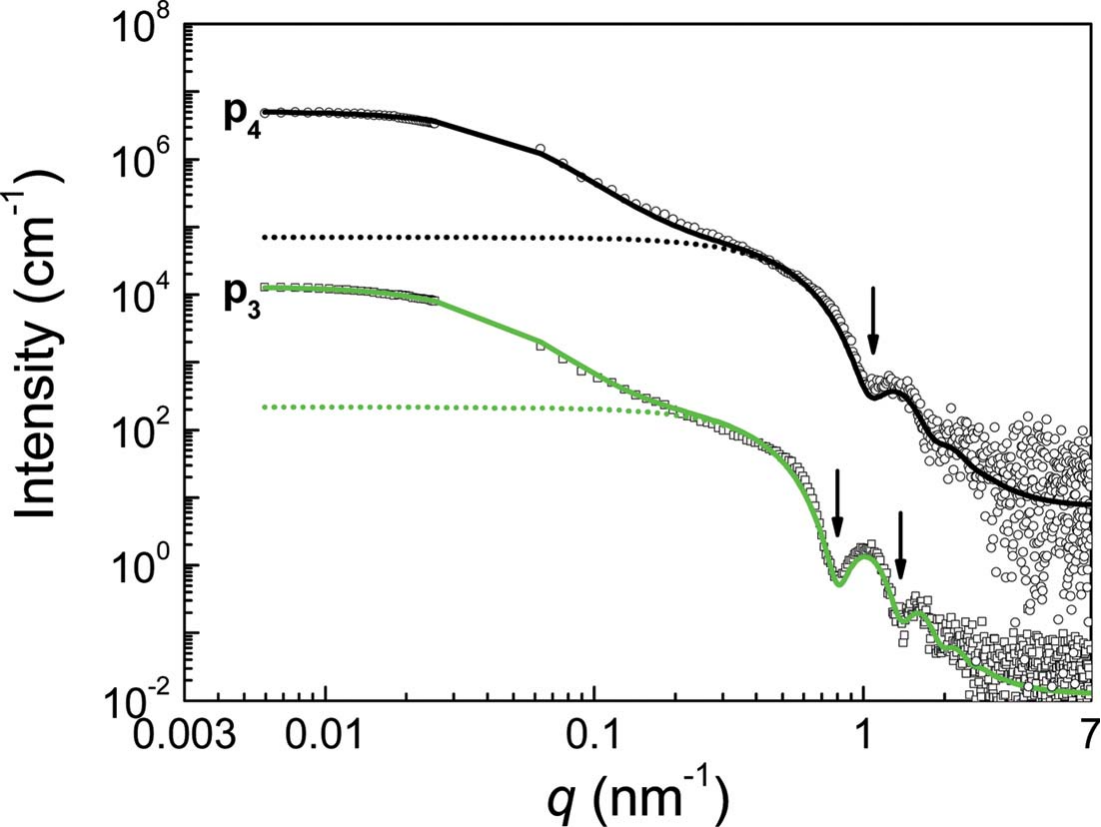
The combined SAXS and SLS curves of multi-core particles p_3_ and p_4_ (squares and circles, respectively). Data of p_4_ are multiplied by 10^3^ for better visibility. Curve fits according to equation (1) are given (solid lines). The mass fractal structure factor in equation (4) is used for consideration of the multi-core structure. Minima at *q* = 0.82 nm^−1^ and *q* = 1.38 nm^−1^ for p_3_ and at *q* = 1.03 nm^−1^ for p_4_ are indicated by arrows. The scattering profiles of the particles alone without structure factor are given as dotted lines.

Visual inspection of the scattering curves of p_3_ and p_4_ reveals similar curve shapes as for p_1_ and p_2_ for *q* values larger than about 0.5 nm^−1^, as shown in Fig. 3 ▸. However, the low-*q* part of the SAXS curve between 0.05 and 0.10 nm^−1^ has a steep rise toward zero. This is a clear sign of larger structure motifs present in the sample in significant numbers. To make an estimate of these large structures, the SAXS curves were combined with static light scattering data to obtain additional scattering data in an interval of 0.59 × 10^−2^ ≤ *q* ≤ 2.60 × 10^−2^ nm^−1^. The SLS and SAXS curves have different intensities. The SAXS curve is plotted in absolute units, whereas the SLS curve is plotted in arbitrary units that are normalized to the value of the first point. Additionally, the ranges of the curves do not overlap; there is a gap that makes merging of the data not straightforward. The solution to the problem is to use the capability of *SASfit* to fit multiple data sets simultaneously, which is usually employed for small-angle neutron scattering (Bressler *et al.*, 2015 ▸). The fits are carried out with two independent parameters for the scattering intensities of the two sets; the other parameters are common for both sets of data. The ratio of the intensity parameter is used to scale the SLS curve intensity, so that it matches the intensity of SAXS. Both sets are merged into one, which is fitted again as a single data set to check for the correctness of the procedure.

The combined data of static light scattering and SAXS in Fig. 3 ▸ show a plateau towards zero. Application of Guinier’s law (Guinier & Fournet, 1955 ▸), 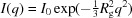, to the light scattering data provides radii of gyration of *R*_g_ = 48 ± 1 nm for p_3_ and *R*_g_ = 44 ± 1 nm for p_4_.

Tentatively, we interpret the larger objects as fractal agglomerates of primary particles. Accordingly, the inter-particle structure factor (Ferretti *et al.*, 1998 ▸; Teixeira, 1988 ▸)
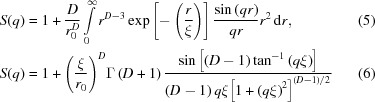
was found appropriate for interpretation of the scattering curves of p_3_ and p_4_ by multiplying the form factor of the primary particle with the fractal structure factor according to equation (1). The results are displayed in Fig. 3 ▸. The parameter *D* in equations (5) and (6) is the fractal dimension, and *r*_0_ is the radius of the individual particles making up the fractal aggregate. Here, we interpret *r*_0_ as the radius of the primary particle core radius plus a shell thickness, which is formed by dimercaptosuccinic acid molecules. ξ is the characteristic size of the fractal or more precisely the cutoff length for the fractal correlation. It represents the distance above which the mass distribution in the aggregate is no longer described by the fractal law. Here the exponential cutoff criterion 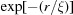 is used.

**Figure 4:**
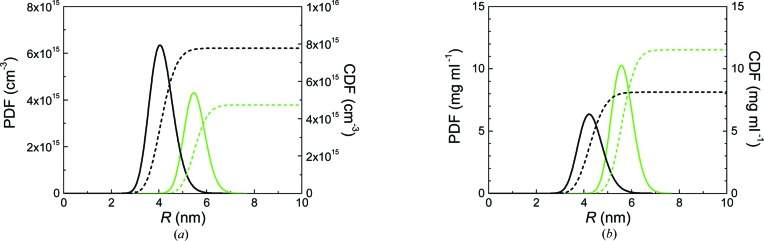
Size distributions of the cores of the primary particles of p_3_ and p_4_ (green and black lines) in number-weighted and volume-weighted form [(*a*) and (*b*), respectively]. Displayed are the partial differential functions (solid lines) and cumulative distribution functions (dashed lines). The number-weighted distributions are given in units of particles per cm^3^. The volume-weighted size distributions in (*b*) are given in units of iron content in mg per ml, calculated on the basis of an Fe_2_O_3_ stoichiometry.

Similarly as in the case of the single-core p_1_, the SAXS curve of multi-core p_3_ shows two sharp minima, at *q* = 0.82 nm^−1^ and *q* = 1.38 nm^−1^, indicating spherical particles with a very narrow size distribution. The SAXS curve of p_4_ has a very similar shape to that of p_3_ at high *q* values (see Fig. 3 ▸). The minimum is blurred, indicating a wider relative size distribution than for p_3_. The position of the minimum at 1.0 nm^−1^ means that the mean core radius is the smallest of the whole series of samples. In the high-*q* range the scattering curve is well reproduced using the form factor of a sphere with lognormal size distribution of moderate width. The best fit results are mean core radii of *R* = 5.5 ± 0.1 nm and *R* = 4.1 ± 0.1 nm for p_3_ and p_4_, respectively. The relative widths of the size distributions are 0.08 ± 0.01 and 0.12 ± 0.02. The scattering data without structure factor contribution are shown separately in Fig. 3 ▸. The corresponding distributions of the particle number densities (PDF and CDF) are shown in Fig. 4 ▸(*a*) (solid and dashed curves, respectively). We calculated the volume-weighted PDF and CDF in terms of iron concentration for comparison (Fig. 4 ▸ *b*). Additionally, the total particle number densities are *N* = (4.7 ± 0.5) × 10^15^ cm^−3^ and *N* = (7.7 ± 0.2) × 10^15^ cm^−3^ (see Fig. 4 ▸ *a*). Calculation of the volume fraction for p_3_ results in φ = (3.3 ± 0.1) × 10^−3^, which corresponds to a total iron oxide concentration of *c* = 16.3 ± 0.2 mg ml^−1^ and a total iron content of 11.4 ± 0.2 mg ml^−1^. Similarly, the volume fraction of p_4_ is φ = (2.2 ± 0.1) × 10^−3^, corresponding to a total iron oxide concentration of *c* = 11.2 ± 0.2 mg ml^−1^ and a total iron content of 7.9 ± 0.1 mg ml^−1^.

The best fit was achieved with a fractal dimension of *D* = 2.9 ± 0.1 for p_3_ and 2.8 ± 0.1 for p_4_. Therefore, the fractal dimensions of p_3_ and p_4_ are the same within the experimental accuracy. Such large values of *D* close to 3 represent compact mass fractal structures. The finding of high fractal dimensions indicates that both multi-core particles are produced by a reaction-limited colloidal aggregation that displays *D* values > 2 (Lin *et al.*, 1989 ▸). The values for ξ are 22 ± 2 nm for p_3_ and 18 ± 2 nm for p_4_. The radii *r*_0_ are 9.3 ± 0.2 nm and 6.8 ± 0.2 nm. The difference of *r*_0_ and *R* can be interpreted as the thickness of the organic coating of the particles. This assumption results in estimates of 3.9 ± 0.2 nm for the thickness of the organic coating of p_3_ and 2.7 ± 0.2 nm for p_4_. These values indicate a thicker coating for p_3_ than for p_4_. The presence of this coating, which is obviously much thicker than a monolayer thickness of the stabilizer, suggests also that the primary particles are isolated from each other by the organic material of the coating.

**Figure 5:**
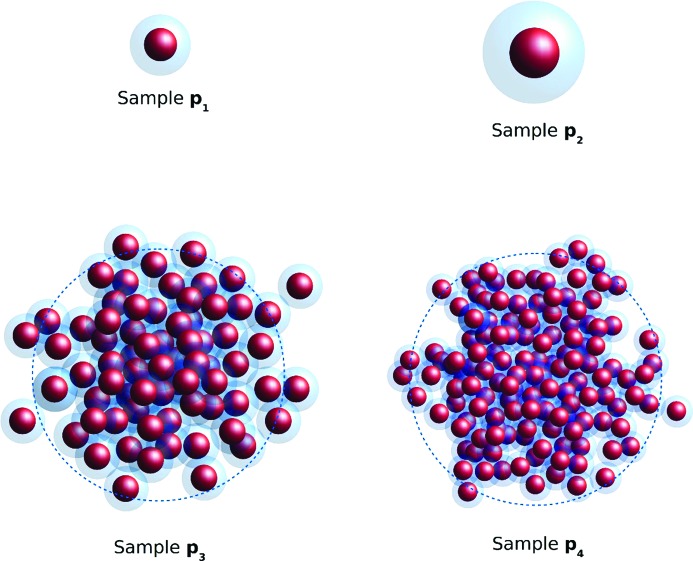
Schematic pictures of single-core (p_1_, p_2_) and multi-core particles (p_3_, p_4_). The primary particles consisting of iron oxide are displayed as solid spheres and their stabilizing shells are transparent blue. The blue dashed lines in the structures of p_3_ and p_4_ indicate the characteristic size of the fractals.

We checked the consistency of our results. For this purpose we used the fact that the radius of gyration can also be determined from the fit parameters (Ferretti *et al.*, 1998 ▸) of the fractal as
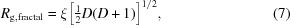
resulting in *R*_g,fractal_ = 52 ± 5 nm for p_3_ and 42 ± 5 nm for p_4_. These values are in agreement with the *R*_g_ values from utilizing Guinier’s law (see Table 1 ▸). The number of primary particles can be estimated as (Teixeira, 1988 ▸)
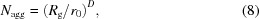
resulting in 117 ± 16 and 186 ± 27 primary particles per aggregate. The fit parameters are summarized in Table 1 ▸ for ease of comparison. A schematic picture of the particles’ structures is shown in Fig. 5 ▸.

## 6. Comparison with TEM   

**Figure 6:**
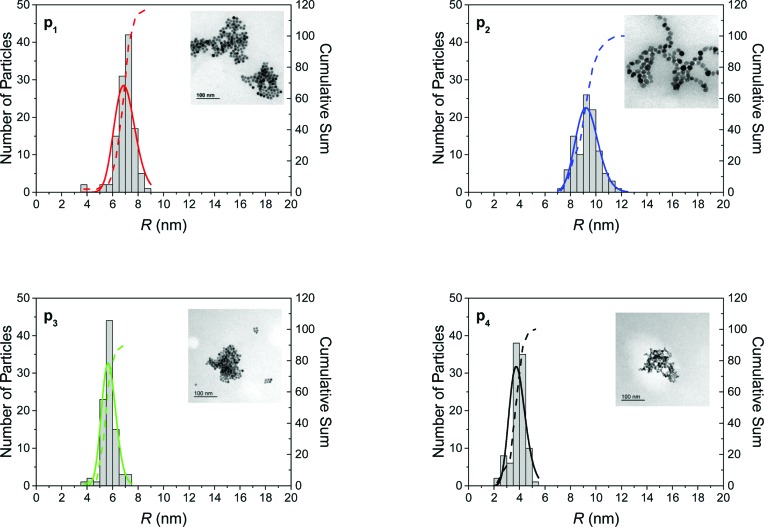
TEM results of particles p_1_, p_2_, p_3_ and p_4_. Histograms represent the number of particles with the radii derived from the pictures in the insets. Displayed are the partial differential functions (solid lines) from lognormal curve fits of the histograms and the cumulative sums from the histograms (dashed lines).

TEM measurements were conducted in order to check our findings from SAXS. The TEM results are summarized in Fig. 6 ▸. The particle radius distributions were derived from the pictures in the insets in the form of histograms. Fits of lognormal functions in PDF and CDF presentation are displayed as solid and dashed lines, respectively. It should be noted that it is hardly possible to distinguish aggregated from non-agglomerated particles by TEM unless it is performed in solution using Cryo-TEM. But such an investigation is difficult to conduct. In addition, it is time-consuming and therefore expensive work to count a large enough number of particles in order to obtain representative radius distributions. Here, the number of particles is of the order of 100 as can be seen from the cumulative sum of the particles (right axes). Nevertheless, fitting of the TEM data with lognormal distributions provides mean radii of 7.0 nm (p_1_), 9.3 nm (p_2_), 5.7 nm (p_3_) and 3.9 nm (p_4_). The relative widths of the radius distributions are 0.12 (p_1_), 0.10 (p_2_), 0.10 (p_3_) and 0.17 (p_4_). Comparison of these TEM results with the summarized values for the primary particles in Table 1 ▸ reveals an excellent agreement of TEM and SAXS values. We therefore conclude that the TEM measurements confirm our results from SAXS.

## 7. Type of iron oxide within the particles   

**Figure 7:**
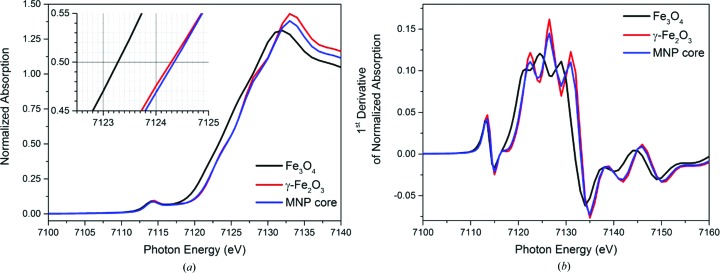
XANES of p_3_, which is representative for all particle cores of the series. The spectral shapes and the peak positions identify the cores unambiguously as γ-Fe_2_O_3_, both in the edge-step normalized spectrum (*a*) and in the first derivative of the absorption spectrum (*b*).

All samples of the series have the same XANES spectrum at the *K* edge of iron as can be seen in Fig. 7 ▸(*a*). The spectrum of magnetic nanoparticle cores exhibits all features of the XANES spectrum of maghemite (Schimanke & Martin, 2000 ▸; Carta *et al.*, 2013 ▸). This is especially recognizable in the first derivative of the XANES spectrum in Fig. 7 ▸(*b*). The main peak and its three distinctive maxima have exactly the same positions as those of the γ-Fe_2_O_3_ reference. This means that no Fe^II^ is present in the sample in detectable amounts. Moreover, the shape of the MNP core spectra follows almost exactly the shape of the maghemite reference spectrum right above the absorption edge. This part of the XANES contains structural information according to the multiple-scattering theory (Rehr & Albers, 2000 ▸). Thus, the iron oxide cores consist only of maghemite phase, which is an inverse spinel structure with Fe^III^ ions only and vacancies in the cation sublattice for charge neutralization. The findings on the internal structure and composition of the particles’ cores are in agreement with the aims of the synthesis route chosen.

## 8. Conclusions   

The X-ray techniques applied here delivered valuable results for the MNP systems under investigation. SAXS gave a thorough characterization of single-core and multi-core MNPs in terms of core size and size distribution, while XANES proved that the desired crystalline structure and oxidation state of the iron ions was indeed achieved. Information on the inner structure of the MNP, as well as on the morphology of the system, *i.e.* core size, size distribution of core aggregates and number of cores within an aggregate, is of extreme interest for use in subsequent calculations and modelling of magnetic properties of these systems. The single-core spherical particles are a starting point for a reference material aiming for the minimization of the effects connected to the shape anisotropy and intra-particle dipolar interactions. The desired narrow size distribution and the lack of significant numbers of aggregated cores can be easily verified by SAXS, as we have shown. A further motivation for this study was to identify spherical multi-core MNPs with the potential to act as a magnetic reference material, with minimal shape anisoptropy and dipolar interaction properties that can be controlled *via* the size of the agglomerates and the number of cores inside such an object. SAXS in combination with SLS delivered estimates of these morphological parameters. This information can be translated into, for example, mean core distances inside the multi-core. This is of great importance for the modelling of such magnetically interacting cores, which often give rise to bulk magnetization behaviour that is hard to understand without such input. This kind of problem would strongly benefit from the use of a USAXS instrument, closing the information gap between SAXS and SLS data.

## Supplementary Material

Supporting information file. DOI: 10.1107/S1600576717002370/aj5284sup1.pdf

## Figures and Tables

**Table 1:** Summary of the particle characteristics derived from scattering methods *R*_g,LS_ is the radius of gyration derived from static light scattering data, *R* is the mean radius of the core of the primary particle, σ/*R* is the relative width of the size distribution, *r*_0_ is the mean radius of a primary particle of core and shell within a multi-core particle, ξ is the cutoff length for the fractal correlation, *D* is the mass fractal dimension of the multi-core particles, and *N*_agg_ is the aggregation number of a multi-core particle. Values in parentheses are uncertainties on the least significant digit.

		Cores		Mass fractal aggregates				
Nanoparticles	*R*_g,LS_ (nm)	*R* (nm)	σ/*R*	*r*_0_ (nm)	ξ (nm)		*D*	*N*_agg_
Single-core particles†								
p_1_	–	6.9 (1)	0.08 (1)	–		–	–	–
p_2_	–	10.5 (1)	0.13 (2)	–		–	–	–

Multi-core particles								
p_3_	48 (1)	5.5 (1)	0.08 (1)	9.3 (2)	22 (2)		2.9 (1)	117 (16)
p_4_	44 (1)	4.1 (1)	0.12 (2)	6.8 (2)	18 (2)		2.8 (1)	186 (27)

† An amount of less than 1% of primary particles is present in aggregates.
