# Supplementary material for: SAXS analysis of single- and multi-core iron oxide magnetic nanoparticles
Source: J Appl Crystallogr. 2017 Mar 14;50(Pt 2):481–8. doi: 10.1107/S1600576717002370 (PMC5377343; doi:10.1107/S1600576717002370)
Supplement: Supplementary file 1 [file j-50-00481-sup1.pdf]

JOURNAL OF
APPLIED
CRYSTALLOGRAPHY

Volume 50 (2017)

Supporting information for article:

SAXS Analysis of Single- and Multi-Core Iron Oxide Magnetic Nanoparticles

Wojciech Szczerba, Rocio Costo, Sabino Veintemillas-Verdaguer, Maria del Puerto Morales and Andreas F. Thünemann

Supporting Information for

SAXS Analysis of Single- and Multi-Core Iron Oxide Magnetic Nanoparticles

Wojciech Szczerba[1,2], Rocio Costo[3], Sabino Veintemillas-Verdaguer[3], Maria del Puerto Morales[3], and Andreas F. Thünemann[1]*

[1] Federal Institute for Materials Research and Testing (BAM), Unter den Eichen 87, 12205 Berlin, Germany

[2] Academic Centre for Materials and Nanotechnology, AGH University of Science and Technology, al. Mickiewicza 30, 30-059 Kraków, Poland

[3] Instituto de Ciencia de Materiales de Madrid, ICMM/CSIC, Sor Juana Ines de la Cruz 3, 28049 Madrid, Spain

[*] AUTHOR EMAIL and PHONE: andreas.thuenemann@bam.de, +493081041610

Comparison of log-normal with normal (Gaussian) size distribution

The results obtained from our SAXS analysis show that the use of a spherical form factor together with a log-normal size distribution of the radii explain the scattering pattern sufficiently. However, it has been reported by O'Grady and Bradbury (Ogrady & Bradbury, 1983) that particle size distributions of ferrofluids can often be assigned either as log-normal or as normal depending on the technique used for particle preparation. For example, the distribution of particles prepared by thermolysis in organic solvents is better represented by a

normal distribution, as opposite to those prepared in aqueous media at low temperatures. In addition, the use of a log-normal size distribution may disguise further agglomerations of the magnetic particles since it asymmetric with a tail toward larger sizes. Therefore, we fitted the data again using a normal distribution and compared the results with the use of a log-normal distribution to clarify whether a distribution type can be preferred.

The resultant curve fits and the size distributions for p_1 to p_4 are shown for comparison in Figure S 1 to Figure S 4. It can be seen that the fit curves represent the data independent on the type of the distribution used. In analogy to the R factor in crystallography (Hamilton, 1965) SASfit also provides an R value as a quality criterion (Bressler *et al.*, 2015). A value of R raging between 0 and about 0.1 indicates a good to acceptable fit. For employing a log-normal radii distribution we determined R values of 0.05 (p_1), 0.12 (p_2), 0.02 (p_2) and 0.03 (p_4). For comparison, the normal distribution results in R values of 0.13 (p_1), 0.05 (p_2), 0.02 (p_2) and 0.03 (p_4). This values are a signature of a good fit quality and led us conclude that the log-normal and the normal distribution are both equally suitable for interpretation of the radii distribution. The reason why both distributions are suitable is that the width of the size distributions in our study is very small. O'Grady and Bradbury estimates that in magnetic analysis the log-normal distribution is favorable and can be employed even when the radii obey a normal distribution if the relative width of the distribution $\frac{\sigma}{R} \leq 0.35$. For these reasons we prefer usage of the log-normal distribution in this study.

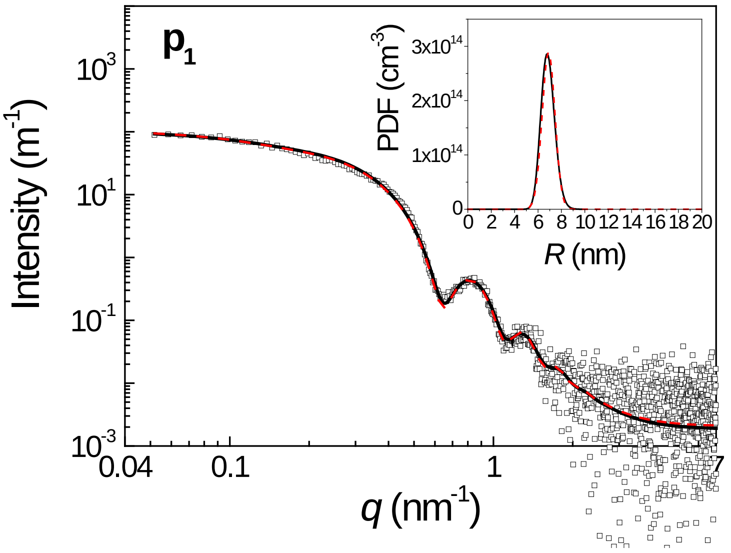

Figure S 1. Comparison of using a log-normal and a normal radii size distribution for fitting the scattering curve of single core particles p_1 (black solid and red dashed line, respectively). The inset shows the corresponding number-weighted radii distributions. Parameters for both curve fits are R = 6.9 ± 0.1 nm and σ/R = 0.08 ± 0.01. The statistics R values of the fits are 0.05 (log-normal) and 0.05 (normal).

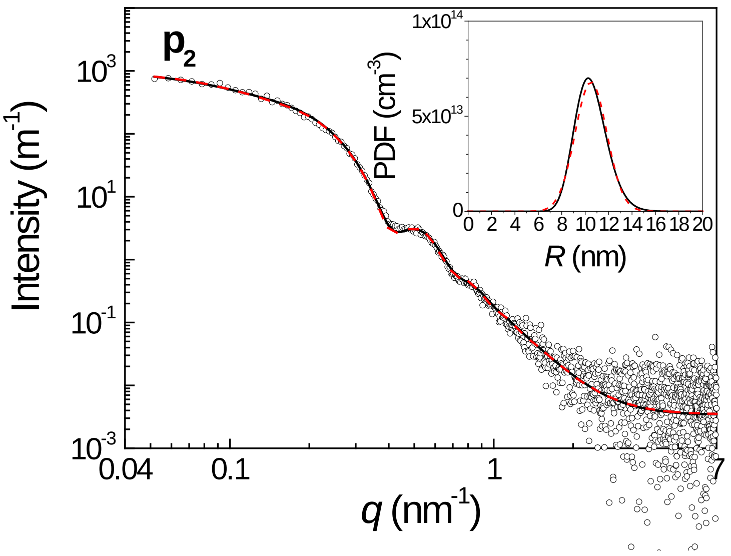

Figure S 2. Comparison of using a log-normal and a normal radii size distribution for fitting the scattering curve of single core particles p_2 (black solid and red dashed line, respectively). The inset shows the corresponding number-weighted radii distributions. Parameters for both curve fits are $R = 10.5 \pm 0.1$ nm and $\sigma/R = 0.13 \pm 0.02$. The statistics R values of the fits are 0.12 (log-normal) and 0.05 (normal).

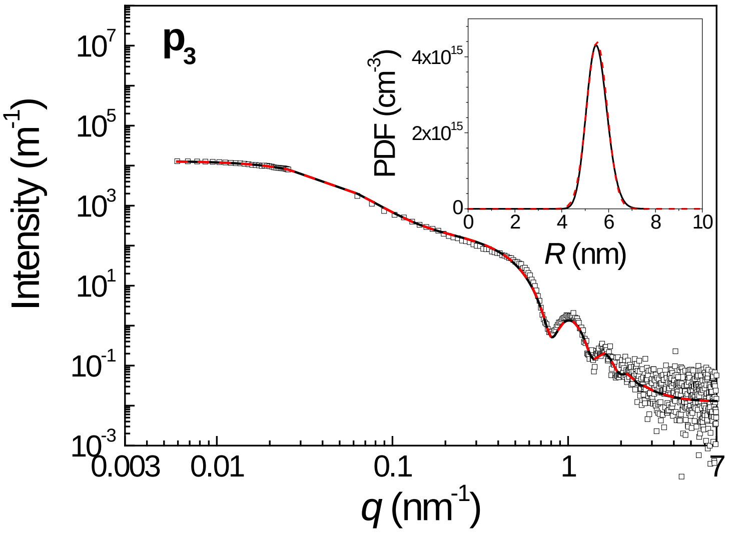

Figure S 3. Comparison of using a log-normal and a normal radii size distribution for fitting the scattering curve of single core particles p_3 (black solid and red dashed line, respectively). The inset shows the corresponding number-weighted radii distributions. Parameters for both curve fits are $R = 5.5 \pm 0.1$ nm and $\sigma/R = 0.08 \pm 0.01$. The statistics R values of the fits are 0.02 (log-normal) and 0.02 (normal).

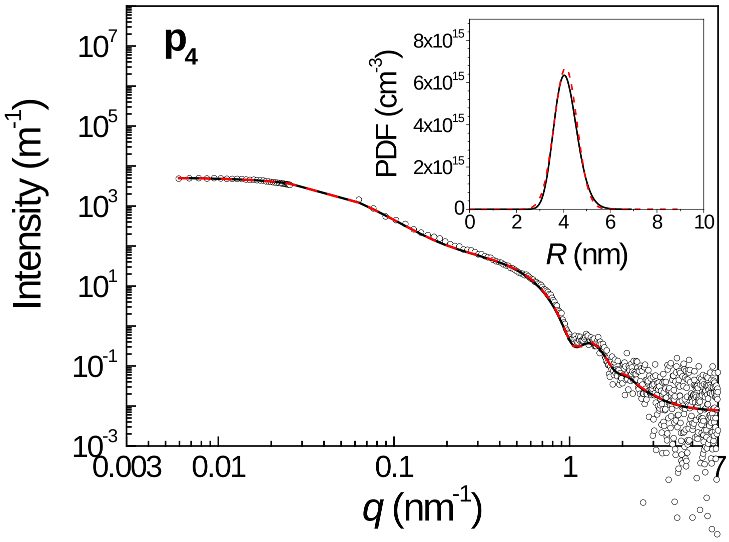

Figure S 4. Comparison of using a log-normal and a normal radii size distribution for fitting the scattering curve of single core particles p_4 (black solid and red dashed line, respectively). The inset shows the corresponding number-weighted radii distributions. Parameters for both curve fits are R = 4.1 ± 0.1 nm and σ/R = 0.12 ± 0.02. The statistics R values of the fits are 0.03 (log-normal) and 0.03 (normal).

References

Bressler, I., Kohlbrecher, J. & Thunemann, A. F. (2015). *Journal of Applied Crystallography* **48**, 1587-1598.

Hamilton, W. C. (1965). *Acta Crystallographica* **18**, 502-510.

Ogrady, K. & Bradbury, A. (1983). *Journal of Magnetism and Magnetic Materials* **39**, 91-94.
